# Ethnic-Guided Soft Tissue Cephalometric Analysis on Lambani Indian Inhabitants for Forensic Facial Reconstruction

**DOI:** 10.7759/cureus.23430

**Published:** 2022-03-23

**Authors:** K Nitya, G S Madhushankari, Keerthi V Narayan, Praveen S Basandi, R Ramya, D Vasumathi

**Affiliations:** 1 Oral and Maxillofacial Pathology, Adhiparasakthi Dental College and Hospital, Chennai, IND; 2 Oral and Maxillofacial Pathology, College of Dental Sciences, Davanagere, IND; 3 Oral and Maxillofacial Pathology, Dr. M.G.R. Educational and Research Institute, Chennai, IND; 4 Oral Biology, Saveetha Dental College and Hospital, Chennai, IND; 5 Epidemiology and Public Health, Thai Moogambigai Dental College and Hospital, Chennai, IND

**Keywords:** ethnic groups, facial reconstruction, soft tissue analysis, lambani inhabitants, kannadiga’s, indian ethnic tribes

## Abstract

Background: Forensic craniofacial reconstruction is a combination of both scientific technique and artistic skill that assist facial soft tissue approximation on the skull to obtain an image of an individual that varies in the different ethnic groups depending on genetic and environmental factors such as soft tissue norms.

Objectives: The present study was aimed to evaluate the soft tissue norms for Lambani Indian tribes spread across the state of Karnataka in India and compare them with the local inherent ethnolinguistic Kannadiga population.

Material and methods: Forty healthy individuals encompassing 20 Lambanis and 20 Kannadigas were selected using demographic information. Lateral cephalograms obtained were analyzed for various soft tissue landmarks that include facial angle, upper lip curvature, skeletal convexity, H angle, nose tip to H-line, upper sulcus depth, lower sulcus depth, upper lip thickness, upper lip strain, lower lip to H line, soft tissue chin thickness, and glabella.

Results: It was observed that glabella thickness, upper sulcus depth, and lower lip to H line showed a significant difference between Lambani and Kannadiga populations. Lambani’s have a higher facial angle than the Kannadiga group though not statistically significant. Gender-wise comparison had shown a significant difference in variables on upper sulcus depth, glabella among females, and lower lip to H line, glabella among males.

Conclusion: The differences obtained between the two ethnic groups in this study clearly suggest the need for separate soft tissue thickness norms for distinctive populations that could be vital in the facial reconstruction of an individual in the field of forensic investigation to narrow down the identification process.

## Introduction

A forensic facial reconstruction or Forensic facial approximation is the process of re-creating the face of an individual whose identity is often not known or clearly appreciable, from their skeletal remains [1]. It is often considered as a reliable tool for recognition in forensic investigations when the routine channels of inquiry such as crime scenes, missing person files and dental records assessment may have already been pursued with limited success [2]. Craniofacial reconstruction is a branch of forensic odontology that has opened a new dimension in the field of identification from skeletal remains in scenarios where the recognition is crucial for medico-legal and investigatory purposes [3]. It is a combination of both scientific method and artistic skill, which can be used to reconstruct the soft tissue onto the skull to obtain an image of an individual. Various techniques currently available for facial reconstruction include two-dimensional Imaging, three-dimensional Imaging, and superimposition method using soft tissue thickness (STT) [4]. In order to obtain an accurate facial STT computed tomography (CT), magnetic resonance imaging (MRI), ultrasonogram (USG), and radiographs have been widely used in recent years according to their consistency and efficiency [5].

Producing a facial from the skull and its remnants is always a challenging task due to its reliability on the relationship between soft tissue covering the skull and underlying bony features along with several factors such as genetic and environmental influences. To achieve this, STT norms for different ethnic groups have been established using Cephalometric analysis in several studies and concluded that there are significant differences amongst various groups [6,7]. However, very few literature studies were available on facial reconstruction soft tissue analysis in the state of Karnataka in India despite multivariate ethnicity across the state. Hence, our study is an attempt to evaluate and estimate STT norms for a Lambanis, also called Lambadis or Banjaras, a nomadic inhabitant tribes spread across the state of Karnataka and compare their STT norms with local inherent Kannadiga’s, an ethno-linguistic Karnataka population group.

## Materials and methods

**Figure 1:**
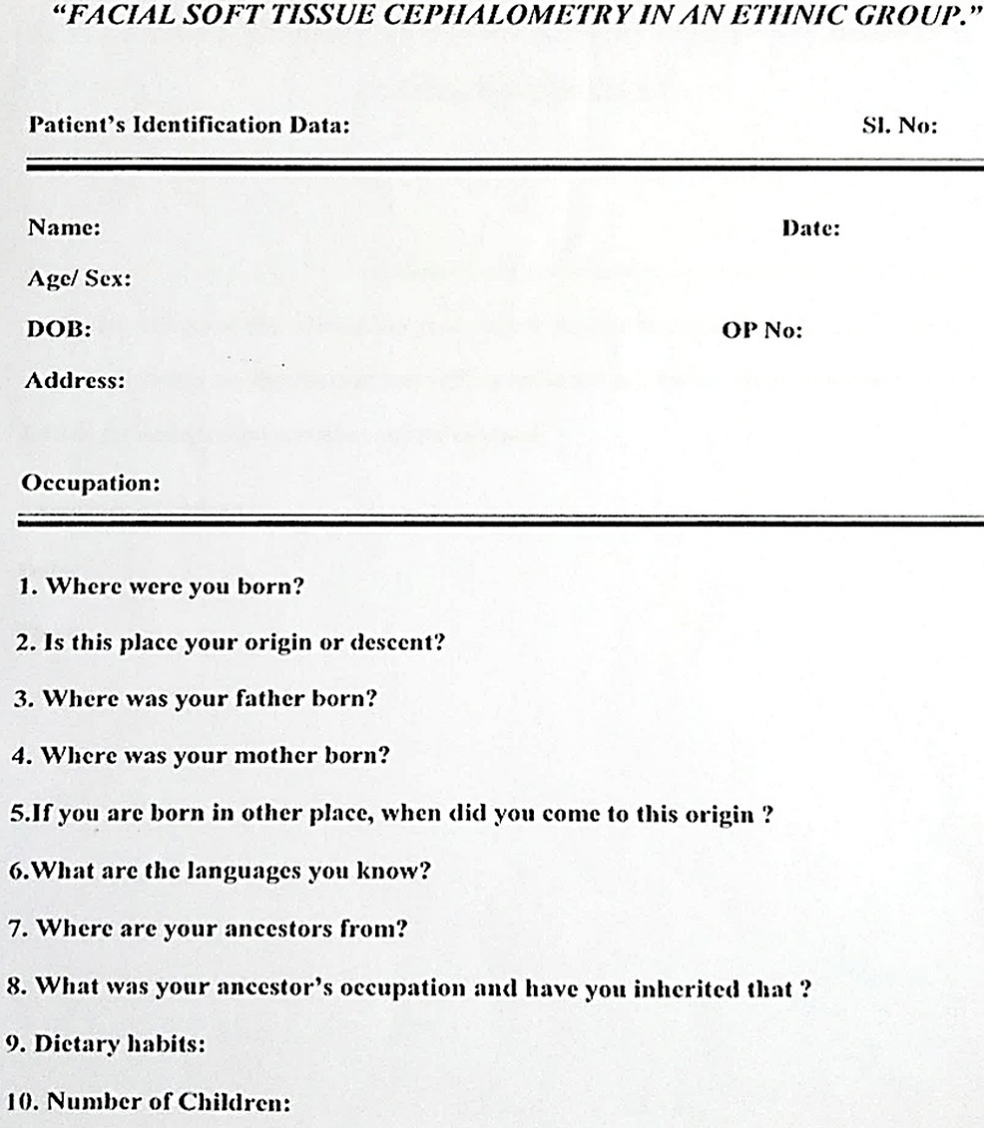
Personal identification datasheet obtained to confirm their origin, ethnicity, or descent

Forty healthy individuals comprising of 20 Lambanis and 20 Kannadigas of age group between 20 to 30 years were selected for this population-based cephalometric study. An equal number of males and females were included in both groups to ensure appropriate distribution of the sample. Individuals less than 18 years of age, individuals from inter-ethnic marriages, and those who had a previous history of maxillofacial trauma were excluded from the study to eliminate the possible role of interethnic influences, growth factors, and environmental associated changes. Demographic and personal Identification data was obtained to confirm their ethnicity and origin or descent (Figure 1). Ethical clearance was obtained from the Institutional Ethical Committee and informed consent was taken in their local language from all the participants and assured that their participation was purely voluntary along with approval for clinical examination and Cephalograms. The lateral cephalograms of each individual were taken with the help of the x-ray apparatus Planmeca Proline XC with Dimax 3. The individual’s head was oriented to natural head position, the teeth are placed in centric occlusion, and lips are well positioned in contact. The cephalogram films were traced and also viewed by all the investigators of the study to avoid inter-observer variability.

**Figure 2:**
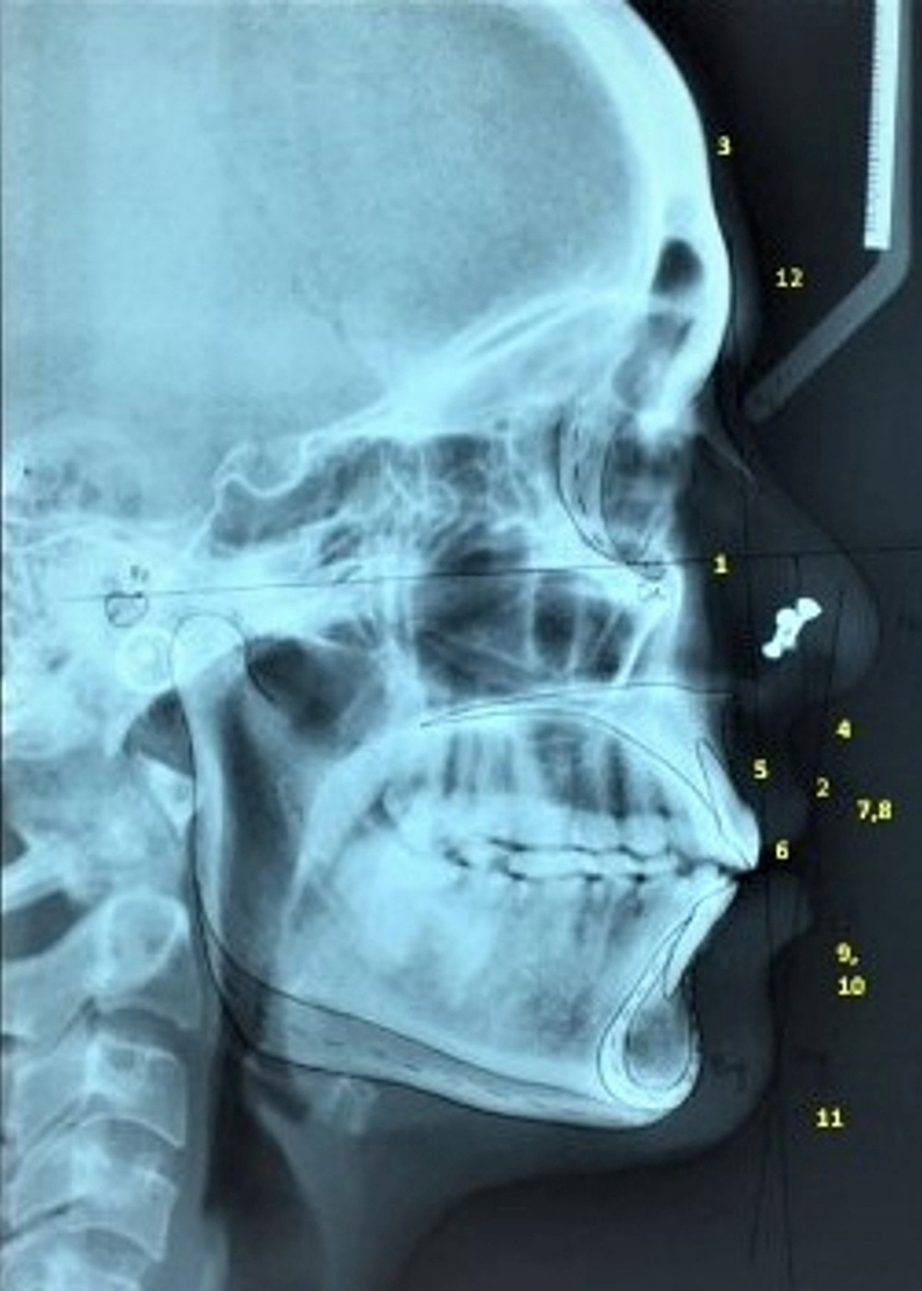
Cephalometric image showing the tracing of soft tissue landmarks in Lambani group 1. Facial angle; 2. Upper lip curvature; 3. Skeletal convexity; 4. H-line angle (Holdaway angle); 5. Nose tip to H line; 6. Upper sulcus depth; 7. Upper lip thickness; 8. Upper lip strain; 9. Lower lip to H line; 10. Lower sulcus depth; 11. Soft tissue chin thickness; 12. Glabella thickness.

**Figure 3:**
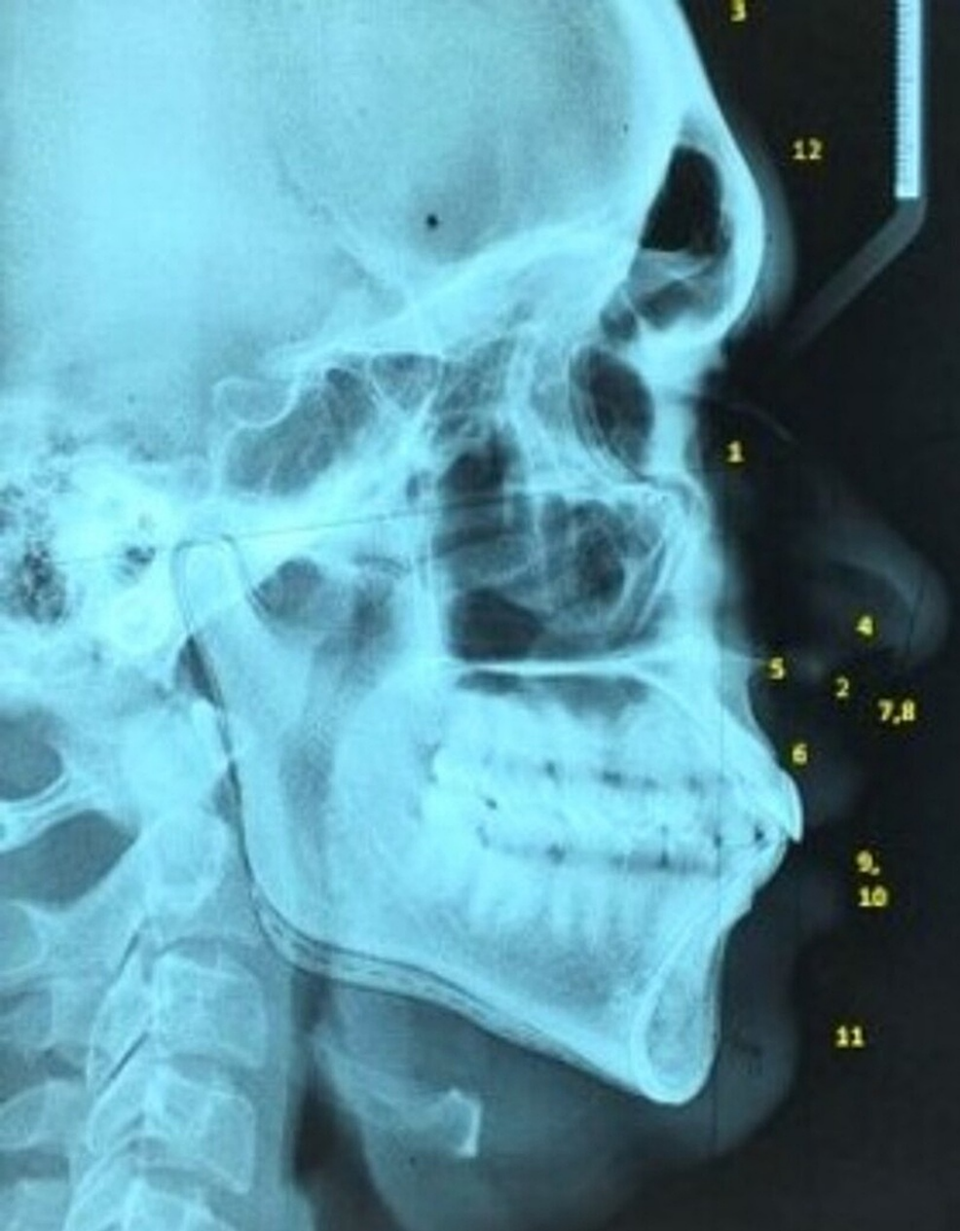
Cephalometric image showing the tracing of soft tissue landmarks in Kannadiga group 1. Facial angle; 2. Upper lip curvature; 3. Skeletal convexity; 4. H-line angle (Holdaway angle); 5. Nose tip to H line; 6. Upper sulcus depth; 7. Upper lip thickness; 8. Upper lip strain; 9. Lower lip to H line; 10. Lower sulcus depth; 11. Soft tissue chin thickness; 12. Glabella thickness.

A total of 12 soft tissue cephalometric points were selected based on their clinical importance suggested in previous literature studies and STT was traced on acetate sheet using 3H pencil measured manually by the operator. The various soft tissue landmarks evaluated in the present study are as follows: facial angle (F-angle), upper lip curvature (ULC), skeletal convexity (SKC), H angle (Holdaway angle), Nose tip to H-line (SNH), Upper sulcus depth (USD), lower sulcus depth (LSD), upper lip thickness (ULT), upper lip strain (ULS), lower lip to H line (LLH), soft tissue chin thickness (STCT), and Glabella (Figures 2, 3). Data analysis was done using SPSS 21.0 (IBM Corp., Armonk, NY, USA) and the statistical significance was fixed at P<0.05. Kolmogorov-Smirnov test was carried out to confirm the normality of the distribution. Descriptive statistics (mean, standard deviation) was calculated for the cephalometric variables. The t-test was conducted to compare the mean cephalometric variables of the two study population groups between male and female population.

## Results

A total of 40 participants consisting of 20 each in the Kannadigas and Lambanis group were selected. An equal number of male and female participants were included in both groups. There was a significant difference in Cephalometric variables like USD, LLH, and Glabella between Lambani and Kannadiga population groups (Table 1). The ULT value was higher and the H-angle value was lower among Lambani, but the difference was not statistically significant (Table 2). When the Cephalometric variables were compared between females of the two groups, there was a significant difference in USD and glabella. There was a slight difference in values with F angle, ULT, and ULS variables, but there was no significant difference (Table 3).

**Table 1:** T-test analysis of cephalometric variables between the groups Facial angle (F-angle); Upper lip curvature (ULC); Skeletal convexity (SKC); Holdaway angle (H-angle); Nose tip to H-line (SNH); Upper sulcus depth (USD); upper lip thickness (ULT); upper lip strain (ULS); lower lip to H line (LLH); lower sulcus depth (LSD); soft tissue chin thickness (STCT) *p<.05 - Significant; N - Number of responses

Variables	Group	N	Mean	Standard Deviation	P-value
Facial angle (F-angle)	Lambani	20	89.16	2.758	.665
	Kannadiga	20	88.75	3.323	.668
Upper lip curvature (ULC)	Lambani	20	3.64	1.236	.164
	Kannadiga	20	4.58	2.825	.182
Skeletal convexity (SKC)	Lambani	20	2.66	1.665	.182
	Kannadiga	20	3.35	1.623	.181
H-angle	Lambani	20	14.18	3.476	.185
	Kannadiga	20	16.25	6.206	.199
Nose tip to H-line (SNH)	Lambani	20	6.48	3.813	.340
	Kannadiga	20	5.25	4.423	.344
Upper sulcus depth (USD)	Lambani	20	5.36	4.006	.004*
	Kannadiga	20	8.58	2.701	.004*
upper lip thickness (ULT)	Lambani	20	15.30	3.884	.081
	Kannadiga	20	13.10	4.051	.081
upper lip strain (ULS)	Lambani	20	2.86	2.305	.193
	Kannadiga	20	3.98	3.105	.200
lower lip to H line (LLH)	Lambani	20	2.59	1.161	.044*
	Kannadiga	20	3.68	2.123	.049*
lower sulcus depth (LSD)	Lambani	20	5.20	3.195	.797
	Kannadiga	20	5.00	1.589	.792
soft tissue chin thickness (STCT)	Lambani	20	12.61	2.273	.513
	Kannadiga	20	12.05	3.220	.520
Glabella	Lambani	20	5.95	0.722	.000*
	Kannadiga	20	4.63	0.767	.000*

**Table 2:** T-test for equality of means analysis of cephalometric variables between the groups Facial angle (F-angle); Upper lip curvature (ULC); Skeletal convexity (SKC); Holdaway angle (H-angle); Nose tip to H-line (SNH); Upper sulcus depth (USD); upper lip thickness (ULT); upper lip strain (ULS); lower lip to H line (LLH); lower sulcus depth (LSD); soft tissue chin thickness (STCT) *p<.05 - Significant; Sig - Significance; Std - Standard; N - Number of responses

Variables	T-test for equality of means	Sig. (2-tailed)	Mean difference	Std. error difference
Facial angle (F-Angle)	Equal variances assumed	.665	.409	.939
	Equal variances not assumed	.668	.409	.947
Upper lip curvature (ULC)	Equal variances assumed	.164	-.939	.662
	Equal variances not assumed	.182	-.939	.684
Skeletal convexity (SKC)	Equal variances assumed	.182	-.691	.508
	Equal variances not assumed	.181	-.691	.508
H-angle	Equal variances assumed	.185	-2.068	1.534
	Equal variances not assumed	.199	-2.068	1.573
Nose tip to H-line (SNH)	Equal variances assumed	.340	1.227	1.271
	Equal variances not assumed	.344	1.227	1.280
Upper sulcus depth (USD)	Equal variances assumed	.004*	-3.211	1.065
	Equal variances not assumed	.004*	-3.211	1.046
upper lip thickness (ULT)	Equal variances assumed	.081	2.195	1.225
	Equal variances not assumed	.081	2.195	1.227
upper lip strain (ULS)	Equal variances assumed	.193	-1.111	.839
	Equal variances not assumed	.200	-1.111	.851
lower lip to H line (LLH)	Equal variances assumed	.044*	-1.084	.521
	Equal variances not assumed	.052*	-1.084	.535
lower sulcus depth (LSD)	Equal variances assumed	.797	.205	.791
	Equal variances not assumed	.792	.205	.768
soft tissue chin thickness (STCT)	Equal variances assumed	.513	.564	.854
	Equal variances not assumed	.520	.564	.868
Glabella	Equal variances assumed	.000*	1.330	.230
	Equal variances not assumed	.000*	1.330	.230

**Table 3:** T-test analysis of cephalometric variables between the groups among the female population Facial angle (F-angle); Upper lip curvature (ULC); Skeletal convexity (SKC); Holdaway angle (H-angle); Nose tip to H-line (SNH); Upper sulcus depth (USD); upper lip thickness (ULT); upper lip strain (ULS); lower lip to H line (LLH); lower sulcus depth (LSD); soft tissue chin thickness (STCT) *p<.05- Significant; N- Number of responses

Variables	Group	N	Mean	Standard deviation	P-value
Facial angle (F-Angle)	Lambani	10	88.65	3.292	.751
	Kannadiga	10	89.20	4.290	.752
Upper lip curvature (ULC)	Lambani	10	3.85	1.107	.117
	Kannadiga	10	5.20	2.348	.124
Skeletal convexity (SKC)	Lambani	10	3.05	1.739	.671
	Kannadiga	10	3.40	1.883	.671
H-angle	Lambani	10	15.15	3.283	.982
	Kannadiga	10	15.20	6.197	.982
Nose tip to H-line (SNH)	Lambani	10	4.95	3.609	.726
	Kannadiga	10	4.30	4.498	.726
Upper sulcus depth (USD)	Lambani	10	4.95	1.499	.000*
	Kannadiga	10	9.35	2.604	.000*
upper lip thickness (ULT)	Lambani	10	14.45	3.270	.319
	Kannadiga	10	12.70	4.296	.320
upper lip strain (ULS)	Lambani	10	2.25	1.620	.291
	Kannadiga	10	3.15	2.055	.292
lower lip to H line (LLH)	Lambani	10	3.05	1.165	.339
	Kannadiga	10	3.95	2.650	.344
lower sulcus depth (LSD)	Lambani	10	5.00	3.742	.879
	Kannadiga	10	5.20	1.687	.880
soft tissue chin thickness (STCT)	Lambani	10	12.05	1.641	.377
	Kannadiga	10	12.80	2.044	.378
Glabella	Lambani	10	5.90	.738	.003*
	Kannadiga	10	4.74	.782	.003*

On the other hand, cephalometric variables compared between males of the two groups showed variables like LLH, glabella were significantly different among males (Table 4). Similarly, the variable H angle value was higher among Lambani females than males, but there was no significant difference. Overall gender wise comparison had shown that there was significant difference in variables USD, glabella among females and LLH, glabella among males between the study populations.

**Table 4:** T-test analysis of cephalometric variables between the groups among the male population Facial angle (F-angle); Upper lip curvature (ULC); Skeletal convexity (SKC); Holdaway angle (H-angle); Nose tip to H-line (SNH); Upper sulcus depth (USD); upper lip thickness (ULT); upper lip strain (ULS); lower lip to H line (LLH); lower sulcus depth (LSD); soft tissue chin thickness (STCT) *p<.05 - Significant; N - Number of responses

Variables	Group	N	Mean	Standard deviation	P-value
Facial angle (F-Angle)	Lambani	10	89.58	2.285	.190
	Kannadiga	10	88.30	2.111	.187
Upper lip curvature (ULC)	Lambani	10	3.46	1.356	.636
	Kannadiga	10	3.95	3.236	.662
Skeletal convexity (SKC)	Lambani	10	2.33	1.600	.153
	Kannadiga	10	3.30	1.418	.149
H-angle	Lambani	10	13.38	3.562	.083
	Kannadiga	10	17.30	6.360	.105
Nose tip to H-line (SNH)	Lambani	10	7.75	3.634	.374
	Kannadiga	10	6.20	4.367	.383
Upper sulcus depth (USD)	Lambani	10	5.71	5.340	.275
	Kannadiga	10	7.80	2.700	.252
Upper lip thickness (ULT)	Lambani	10	16.00	4.343	.178
	Kannadiga	10	13.50	3.979	.175
Upper lip strain (ULS)	Lambani	10	3.38	2.715	.320
	Kannadiga	10	4.80	3.824	.338
Lower lip to H line (LLH)	Lambani	10	2.21	1.054	.043*
	Kannadiga	10	3.40	1.524	.046*
Lower sulcus depth (LSD)	Lambani	10	5.38	2.821	.572
	Kannadiga	10	4.80	1.549	.553
soft tissue chin thickness (STCT)	Lambani	10	13.08	2.670	.230
	Kannadiga	10	11.30	4.057	.252
Glabella	Lambani	10	6.00	.739	.000*
	Kannadiga	10	4.51	.775	.000*

## Discussion

Forensic facial reconstruction is an attempt to produce the likeliness of the facial features of an individual based on the characteristics of the skull for the purpose of individual identification. The face of an individual usually comprises of different features and uniqueness which is of great importance in identification and also in recognition of an individual. Manual methods of facial reconstruction require a great deal of anatomic knowledge as well as artistic modeling exposure. In recent years two-dimensional, three-dimensional, computer-aided techniques have been attempted to simplify the technique and lessen the time required for the same [4]. Earlier studies suggested that facial STT can be measured using needle puncturing on the cadavers but were not accurate due to dehydration and shrinkage of tissue [8,9]. This was overcome by measuring the soft tissue depth at various points using CT, MRI, USG, and Lateral cephalograms [5]. At present, studies on facial SST are been increasing and they also differ from each other by the imaging technique and the selected population sample. One of the most accurate measurements is obtained from CT, MRI but they belong to invasive technique due to high radiation. Lateral cephalograms studies are also being done and the Cephalometric norms for different ethnic and racial groups have been established previously [10].

Lambanis or Lamani/Lambadi is the biggest ethnic tribal group in India. The name was derived from the Sanskrit word “banaj/vanjiya” meaning trading, because of their main and age-old vocation of transportation of food grains and salt. The Romans are all over the world and more densely populated in the European countries that are the members of Nomadic group and are originated from the Indian sub-continent. Few literature studies believe these nomadic tribes are immigrants who came from Afghanistan to Rajasthan and have now spread themselves across several states of India including Karnataka, Andhra Pradesh, and Maharashtra [11,12]. These Lambanis have been called different names in different parts of the country. In Karnataka, they are referred to as Lambani. Although their origin, history, culture, language are very vast and studies for facial reconstruction in these populations are very few. However, the present study attempted to compare the soft tissue depths available on the lateral cephalograms with the actual soft tissue profile of the subject’s photograph. As there was a significant similarity between the two groups, this contemporary study suggested that the soft tissue depths determined in this manner could be used to reconstruct the profile of the subject where only a skull is available.

In the present study, glabella thickness, upper sulcus depth, and lower lip to H line show a significant difference between Lambani and Kannadiga populations. Lambanis have a higher facial angle than the Kannadiga group which is not statistically significant. The results of the present study show that there is a significant facial SST in glabella, upper sulcus depth, and lower lip to H line which was not significant in the previous study done on the Lambada population [12]. Lateral cephalograms study done on Haryana population showed significant difference at certain points like sub-nasale, pro-nasale, Labrale superior, and inferior [1]. Facial soft tissue cephalometric norms in Central Indian population showed that significant differences were observed between the Central Indian and White populations. Also significant in sexual dimorphism was noted between males and females of the Central Indian population [13,14]. A comparative study done on Lambada population showed that skeletal convexity and H angle were significantly correlated in female population [12]. Sinojiya et al. reported a significant difference between males and females of Mahabubnagar population. Males have thicker soft tissue structures compared to females [15]. Several parameters are significantly different among both the ethnicity group in the present study. This variation in the features of soft-tissue analysis might also have influenced by several factors such as genetic, diet modifications, periodic and seasonal factors and their inhabitant lifestyle. These findings also revealed that soft tissue norms varied according to gender that is often considered as critical prerequisites for accurate personal identification in crime and medico-legal situations in the field of forensic investigation.

Limitations of the study

There are certain limitations to our study. First being the role of environmental factors like diet, way of living, unfamiliar marriage patterns such as intermarriages may have influenced few parameters considered in this ethnic-based assessment. Second, this assessment is limited to Holdaway’s analysis parameters established for a Caucasian population with few parameters of considerable significance. Few other limitations include inherent biases of analysis, smaller sample size, as well as errors during the cephalometric assessment.

## Conclusions

From the results of the present study, within the limitations, it can be concluded that the Lambani population had prominent glabella, upper sulcus depth, and lower lip to H line when compared to the Kannadiga population. The differences obtained between the ethnic groups clearly suggest the need for separate STT norms for distinctive populations that could be vital in the facial reconstruction of an individual whose identity is often not known or clearly appreciable, from their skeletal remains in the field of forensic investigation. Hence, it is important to compare between different ethnic groups, which help the forensic investigations to narrow down the identification process.
